# Urethritis caused by nongroupable *Neisseria meningitidis* ST-11 and ST-11026 in China

**DOI:** 10.1016/j.nmni.2025.101671

**Published:** 2025-11-19

**Authors:** Rui Jin, Xiaoyu Zhu, Yanhong Zhao, Biyu Yin, Shaochun Chen

**Affiliations:** a Hospital for Skin Diseases, Institute of Dermatology, Chinese Academy of Medical Sciences & Peking Union Medical College, Nanjing, 210042, China; b School of Public Health, Nanjing Medical University, Nanjing, 211166, China

**Keywords:** *Neisseria meningitidis*, Urethritis, Sexually transmitted infections, Antimicrobial resistance, China

Dear Editor,

*Neisseria meningitidis* (Nm) typically colonizes the human nasopharynx and is a major cause of invasive meningococcal disease (IMD). However, Nm has expanded its niche. The United States reported an outbreak of urethritis caused by nongroupable Nm of sequence type 11 (ST-11). These strains form a distinct clade termed the US *N. meningitidis* urethritis clade (US_NmUC) [1]. The US_NmUC isolates have acquired multiple genes from *Neisseria gonorrhoeae* (Ng), enhancing their adaptation to the urogenital tract [1]. This emerging pathogen has spread to Europe [2] and Asia [3], where it further evolved during transmission to form the NmUC-B subclade. Based on whole-genome sequencing, we identified five urethritis-associated Nm isolates belonging to ST-11 and ST-11026 in China.

To assess the accuracy of routine diagnostic methods in distinguishing urethritis-associated Nm from Ng, we used MALDI-TOF MS, VITEK 2 NH/API NH, and commercial Ng nucleic acid amplification tests (NAATs) (Supplementary Table S2) to identify the isolates. Except for one isolate that failed to revive, all others were identified as Nm by MALDI-TOF MS and tested negative by Ng NAATs. However, VITEK 2 NH/API NH misidentified NM-NCSTDC01 as Ng, while correctly identifying the remaining isolates. This misidentification was attributable to the loss of maltose utilization, resulting in a carbohydrate utilization profile resembling that of Ng.

Multilocus sequence typing (MLST) analysis showed that NM-NCSTDC01 and NM-NCSTDC02 belonged to ST-11, whereas NM-NCSTDC03–05 (NM-NCSTDC03, NM-NCSTDC04, and NM-NCSTDC05) were identified as ST-11026 (Supplementary Table S1). ST-11026 had previously been reported only in Japan and was the predominant ST among Japanese non-IMD strains [4]. Further phylogenetic analysis revealed that NM-NCSTDC01 and NM-NCSTDC02 clustered within NmUC-B (Fig. 1), a subclade of NmUC that diverged from the original US_NmUC. This subclade contains additional Ng-derived genomic regions [3]. In contrast, NM-NCSTDC03–05 were phylogenetically distinct from the NmUC lineage and more closely related to respiratory Nm isolates, suggesting distinct origins and multiple independent transmission events.

**Fig. 1:**
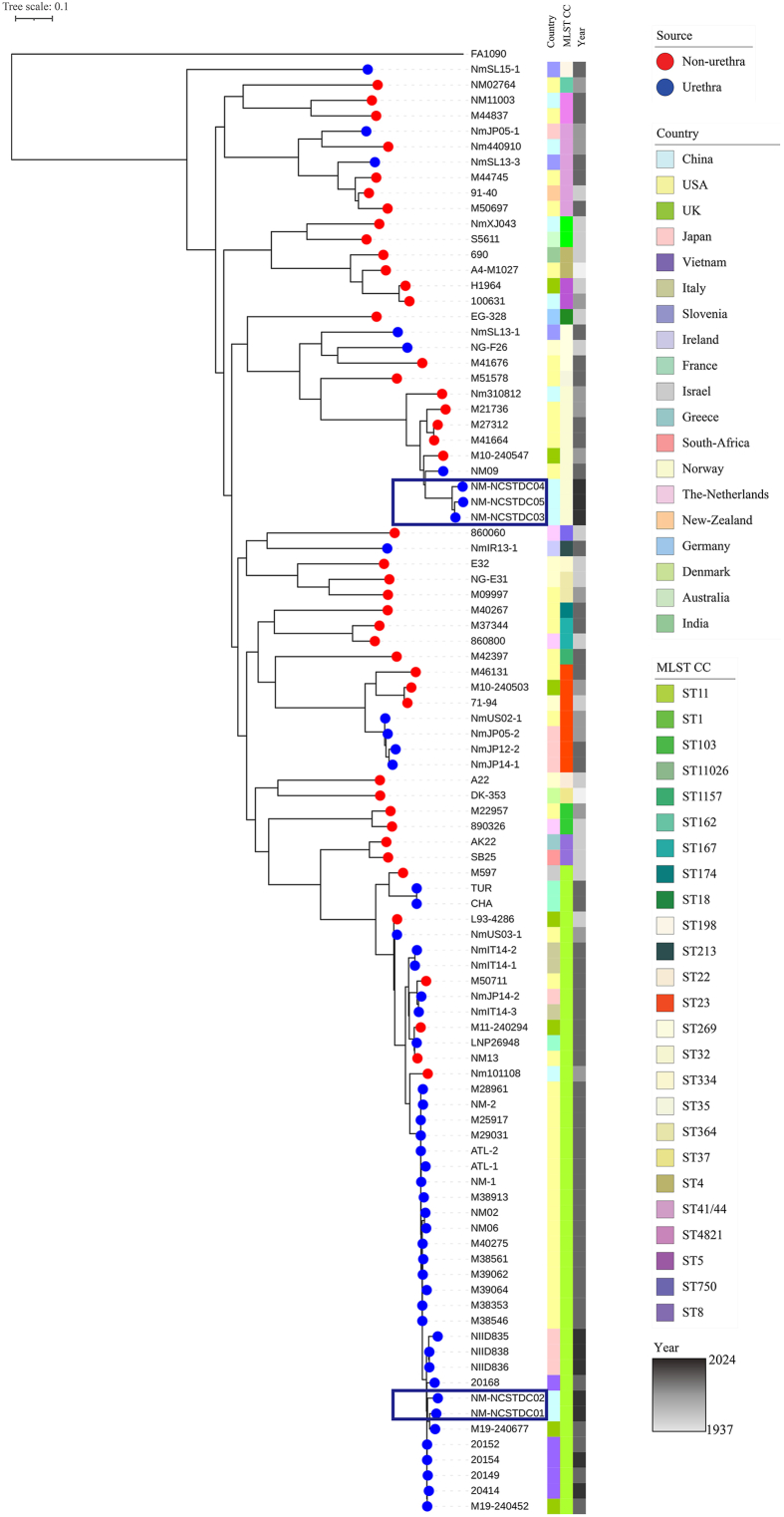
Phylogenetic tree of *Neisseria meningitidis* urethral isolates in China compared with representative clonal complex strains of *Neisseria meningitidis* and ST-11 NmUC strains based on SNPs called from whole genome sequences. *Neisseria gonorrhoeae* FA1090 was used as an outgroup. Blue boxes indicate the strains characterized in this work. Source is indicated by the tip color. Isolates were marked at the first column for Country, the second column for MLST CC, and the third column for year. NmUC, *N. meningitidis* urethritis clade; MLST CC, multilocus sequence typing clonal complex.

Genomic analysis revealed that NM-NCSTDC01 and NM-NCSTDC02 shared key genomic characteristics with the NmUC lineage. These isolates acquired an *aniA***–***norB* locus derived from Ng (Supplementary Table S3), which facilitates colonization in the microaerobic urethral environment [1]. NM-NCSTDC01 and NM-NCSTDC02 were nongroupable, characterized by IS1301-mediated deletion of *cssA/B/C* and partial *csc* genes within the *cps* locus. Furthermore, two additional gonococcal recombination events (NEIS1446–NEIS1442 and NEIS1038) were identified in NM-NCSTDC01 and NM-NCSTDC02 (Supplementary Table S4). In contrast, NM-NCSTDC03–05 displayed distinct genomic characteristics. Their *aniA***–***norB* loci and encoded proteins retained more Nm-like features. Although ST-11026 isolates were also nongroupable, the *cps* locus deletion patterns differed from those of NmUC isolates, as the capsule biosynthesis and transport regions were completely deleted in the ST-11026 isolates. These findings suggest that ST-11026 strains may utilize unidentified mechanisms for adaptation to the urogenital tract.

Antimicrobial susceptibility testing for ceftriaxone, azithromycin, ciprofloxacin, and penicillin was performed using the agar dilution method, with resistance breakpoints interpreted according to the Clinical and Laboratory Standards Institute (CLSI) M100, 43rd edition. These isolates were susceptible to ceftriaxone and azithromycin, but resistant to penicillin and ciprofloxacin. Analysis of resistance determinants (Supplementary Table S5) revealed that NM-NCSTDC01 carried *penA*316, NM-NCSTDC02 carried *penA*1251, and NM-NCSTDC03–05 carried *penA*33. The *penA* alleles identified in our isolates shared substitutions (F504L, A510V, I515V, H541N, and I566V) associated with reduced penicillin susceptibility. Furthermore, all isolates harbored *porB* (NEIS2020) alleles carrying the A121D substitution. For ciprofloxacin resistance, NM-NCSTDC01 carried T91F and D95G mutations in *gyrA*140; NM-NCSTDC02 carried T91F and D95A mutations in *gyrA*9; and NM-NCSTDC03–05 shared *gyrA*376, with a T91I mutation. These mutations have been shown to confer resistance to ciprofloxacin [3].

Urethritis outbreaks caused by distinct Nm sequence types, including ST-11, ST-11026, and the urethritis-associated MenY ST-1466 in Australia [5], highlight the increasing genomic diversity of urethritis-associated Nm and the continuous adaptive evolution for the urethral niche. Another crucial concern is the development of antimicrobial resistance. The coexistence of Nm and Ng within the urethral niche provides a favorable environment for horizontal gene transfer, potentially accelerating the dissemination of antimicrobial resistance determinants in Nm.

In conclusion, we report the emergence of urethritis-associated Nm in China. The increasing genetic diversity and global dissemination of urethritis-associated Nm highlight the need for enhanced surveillance to prevent further dissemination and the continued development of antimicrobial resistance.

## CRediT authorship contribution statement

**Rui Jin:** Conceptualization, Data curation, Formal analysis, Investigation, Validation, Visualization, Writing – original draft, Writing – review & editing. **Xiaoyu Zhu:** Conceptualization, Formal analysis, Investigation, Validation, Writing – review & editing. **Yanhong Zhao:** Investigation, Validation, Writing – review & editing. **Biyu Yin:** Investigation, Validation, Writing – review & editing. **Shaochun Chen:** Conceptualization, Data curation, Funding acquisition, Project administration, Supervision, Writing – review & editing.

## Funding statement

This work was funded by 10.13039/501100016334Jiangsu Provincial Medical Key Laboratory, 10.13039/501100002916Jiangsu Province Capability Improvement Project through Science, Technology and Education (ZDXYS202204). The funders played no role in the study design, data collection and analysis, or the decision to submit the work for publication.

## Declaration of competing interest

The authors declare the following financial interests/personal relationships which may be considered as potential competing interests: Shaochun Chen reports financial support was provided by 10.13039/501100016334Jiangsu Provincial Medical Key Laboratory, 10.13039/501100002916Jiangsu Province Capability Improvement Project through Science, Technology and Education (ZDXYS202204). If there are other authors, they declare that they have no known competing financial interests or personal relationships that could have appeared to influence the work reported in this paper.
